# A 13 year-old boy with post-transplantation lymphoproliferative disorder presenting with obscure gastrointestinal bleeding: a case report

**DOI:** 10.12688/f1000research.3252.1

**Published:** 2014-04-07

**Authors:** Edith Y. Ho, Vijay George, Marjorie McCracken, James W. Ostroff

**Affiliations:** 1 Division of Gastroenterology, Department of Medicine, Case Western Reserve University/Louis Stokes Cleveland Veterans Affairs Medical Center, Cleveland, OH, 44106, USA; 2 Department of Pathology and Laboratory Medicine, University of California San Francisco, CA, San Francisco, 94131, USA; 3 Department of Pediatrics, University of California San Francisco, San francisco, CA, 94131, USA; 4 Division of Gastroenterology, Department of Medicine, University of California San Francisco, San Francisco, CA, 94131, USA

## Abstract

One well recognized and potentially serious complication of chronic immunosuppression in organ transplant recipients is post-transplantation lymphoproliferative disorders (PTLD). This accounts for 20% of all malignancies in transplant recipients, which is four times higher than the general population ^1,2^. The diagnosis of PTLD is often difficult, due to various manifestations resulting in late diagnosis. We report an unusual presentation of PTLD in a pediatric patient where the diagnosis was achieved only after extensive investigation.

## Case presentation

This case involves a 13 year-old Caucasian boy with a history of cystic fibrosis status post deceased donor *EnBloc* combined liver and pancreas transplantation, presenting with gastrointestinal bleeding. His immune suppression regimen after the transplant included tacrolimus 1.5 mg twice daily.

**Figure 1.**
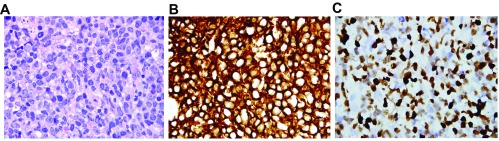
Biopsy of jejunal ulcer. **1A**: Haematoxylin & Eosin stain, 1000x. Large, centroblastic lymphoid cells with hyperchromatic, pleomorphic nuclei and moderate cytoplasm. These tumor cells almost completely efface the jejunal mucosa. Scattered mitotic activity and apopotic debris are present as well. **1B**: Immunohistochemical stain for CD20, 1000x. Diffusely positive membranous staining in the tumor cells is indicative of lymphoid differentiation. In the context of the morphology seen in Figure **1A**, the findings are compatible with a Diffuse Large B-Cell Lymphoma (DLBCL). Per the 2008 World Health Organization (WHO) classification, a lymphoid proliferation that meets the criteria for a high grade malignancy (in this case DLBCL) that occurs in a recipient of a solid organ, bone marrow, or stem cell transplant is diagnostic of a post-transplant lymphoproliferative disorder (PTLD). **1C**: *In situ* hybridization for Epstein-Barr virus encoded RNA (EBER), 1000x. Strong nuclear positivity is seen in the majority of the tumor cells, a finding seen in many monomorphic PTLD cases.

Two years after his transplant, the patient presented to the hospital with “a few days of dark tarry stool”. He did not complain of any nausea, vomiting, or abdominal pain. He denied taking any anti-platelet agents or non-steroidal inflammatory drugs. On admission, the patient was hypotensive with a blood pressure of 70/50 mmHg and tachycardic with a heart rate of 112 beats per minute. His hemoglobin was 7.4 g/dL, which represented a drop from a baseline hemoglobin of 10 g/dL. After fluid resuscitation, the patient was started on pantoprazole continuous infusion at 5 mg/hour and sulcrafate suspension 300 mg four times daily. He then underwent an urgent upper endoscopy, which revealed multiple small prepyloric and duodenal ulcerations without signs of recent hemorrhage. Biopsies of these ulcers showed acute inflammation. A capsule endoscopy was then performed, which showed scattered duodenal erosions and two adjacent erosions in the distal duodenum without stigmata of recent bleeding. During the work-up, the patient continued to have melena, necessitating transfusions every three days. This prompted a second upper endoscopy, which showed complete healing of the prior ulcers. Interestingly, portal hypertensive gastropathy and a gastric varix were also noted. To target bleeding lesions potentially related to portal hypertension, octreotide continuous infusion at 2 mcg/kg/hour was initiated. Unfortunately, his melena persisted. The patient underwent a technetium-labeled red blood cell bleeding scan, where a potential bleeding source “near the anastomosis of the native to transplanted duodenum or proximal jejunum” was identified. A visceral arteriogram was obtained but it failed to detect a bleeding source. Following these radiological tests, a deep enteroscopy was performed. No signs of recent bleed within the suspected proximal hepatic limb of the Roux were detected. In the jejunum, however, there was a 10 mm clean-base, friable ulcer with significant oozing when it was biopsied. The pathological diagnosis of the ulcer was reported as follows: “the morphologic and immunophenotypic features indicate involvement of this patient’s jejunum of a large B-cell lymphoproliferative disorder consistent with diffuse large B-cell lymphoma (DLBCL). These findings thus support the diagnosis of a post-transplantation lymphoid disorder, involving small intestinal mucosa”. Epstein-Barr virus (EBV) was positive in the biopsied sample (See Figure 1).

## Follow-up and outcome

An abdominal MRI performed a few days later demonstrated a mild thickening of the segments of small bowel in the left upper quadrant and portal hypertension. No lymphadenopathy was found in the abdomen and pelvis. The patient was then evaluated by the pediatric oncology service. A bone marrow biopsy reassuringly showed a normocellular marrow. The patient was started on cyclophosphamide, rituximab, and prednisone. His gastrointestinal bleeding eventually stopped a few weeks later.

## Discussion

PTLD are rare but potentially fatal complications of solid organ transplantation ^3^. The overall incidence of lymphoproliferative disorders in the transplant population is approximately 1% at 10 years, which is significantly higher than the general population ^3^. The risk increases with the intensity of induction or rescue immunosuppression, and particularly following monoclonal or polyclonal anti-lymphocyte therapy ^4– 6^. The EBV serostatus of the recipient is another risk factor ^7^, although PTLD can occur in EBV-negative diseases as well. Time post-transplant is also a key factor as the majority of PTLD occurs within the first year post-transplant ^8^. Over half of the patients with PTLD present with extranodal masses ^9^. PTLD can affect many organs, including the gastrointestinal tract, lungs, liver, central nervous system, and the allograft itself. Clinical manifestations vary, ranging from benign polyclonal lymphoproliferation (infectious mononucleosis-type acute illness) to aggressive and disseminated malignant disease. Gastrointestinal features typically include abdominal pain, fever, and bowel perforation in serious cases. There have been case reports describing gastrointestinal bleeding as the initial presentation of PTLD in the pediatric population ^10– 13^, but this is less common. This case highlights the importance of considering the diagnosis of post-transplantation lymphoproliferative disorders in transplant receipts presenting with unexplained gastrointestinal hemorrhage.

## Consent

Informed consent for publication of clinical details and clinical images was obtained from the next of kin.
