# Supplementary material for: A 13 year-old boy with post-transplantation lymphoproliferative disorder presenting with obscure gastrointestinal bleeding: a case report
Source: F1000Res. 2014 Apr 7;3:88. [Version 1] doi: 10.12688/f1000research.3252.1 (PMC4168743; doi:10.12688/f1000research.3252.1)
Supplement: Peer review history

# Peer review: Referee response for version 1

**DOI:** 10.5256/f1000research.3492.r4390

**Published:** 2014-08-04

**Authors:** David Teachey

**Affiliations:** 1 Department of Pediatric Hematology and Oncology, Children's Hospital of Philadelphia, Philadelphia, PA, USA

This is an interesting case report that emphasizes the need to consider the diagnosis of PTLD in post-transplant recipients who present with GI bleeding. It is a well-written report but could be improved by additional details.

 

1. Readers could benefit from a focused discussion on the treatment of PTLD in the post-transplant setting, including reduction in immune suppression, modulation of immune suppression (e.g. transition from a calcineurin inhibitor to sirolimus), single agent therapy with rituximab, or multi-agent therapy with prednisone, cyclophosphamide, and rituximab. In this case based on the pathology, multi-agent chemotherapy was the appropriate treatment, but in other instances less aggressive measures are sometimes used.
2. Additional details including imaging of his extra-abdominal compartment by CT or PET-CT, EBV viral load, and LDH would be helpful. Also, more details on the length and response to treatment would be of interest (was he reimaged or re-scoped?).

I have read this submission. I believe that I have an appropriate level of expertise to confirm that it is of an acceptable scientific standard.

# Peer review: Referee response for version 1

**DOI:** 10.5256/f1000research.3492.r4391

**Published:** 2014-05-07

**Authors:** Patrizia Comoli

**Affiliations:** 1 Pediatric Hematology/Oncology, Fondazione IRCCS Policlinico S. Matteo, Pavia, Italy

This is an interesting case that highlights the importance of considering PTLD in the differential diagnosis of gastrointestinal bleeding occurring after transplantation. The case report is well written, but requires a few minor revisions.

**Article content**

- A few clinical details, important for the diagnosis of PTLD, such as EBV status at transplantation, EBV DNA at diagnosis and at treatment re-evaluation, and LDH at diagnosis, should be inserted.
- Was a PET scan performed to complete PTLD assessment? In order to increase the clinical value of this report, I believe it is important to add data on patient imaging assessment at diagnosis (small bowel MRI and, if performed, total body PET scan, as figure 2 a and b). If PET scan or CT scan were not performed, add a comment on the reasons for not performing imaging assessment.

**References**

- The reference for 2008 World Health Organization (WHO) classification (cited in the legend to Figure 1) ought to be reported.
- Reference 3 relates to stem cell rather than solid organ transplantation. The authors need to replace this reference with a more appropriate one, possibly published within the last 10 years.
- Reference 8 is very outdated. The same author has published a review on the same topic in 2003 ( Opelz & Dohler, 2003), thus the authors should replace the 1993 article with the 2003 review.

I have read this submission. I believe that I have an appropriate level of expertise to confirm that it is of an acceptable scientific standard.
